# Molecular Mechanisms and Immune Regulation in Prostate Cancer: A Review

**DOI:** 10.3390/ijms27167256

**Published:** 2026-08-14

**Authors:** Nigel P. Murray

**Affiliations:** Hospital de Carabineros de Chile, Santiago 7630568, Chile; pmurrayn@hoscar.cl

**Keywords:** prostate cancer, immunosuppression, exosomes, micrometastasis, dormancy

## Abstract

Prostate cancer is formed of a heterogeneous population of cancer cells with different biological properties. They initially form a small part of the normal stromal microenvironment but are able, through cell-to-cell contact and via exosomes, small nanoparticles containing DNA, mRNA, microRNA, long non-coding RNA, enzymes, and chemokines and cytokines, to transform the normal stromal cells into tumour-associated cells to create an immunosuppressive environment as well as inhibit the antitumour immune response. Matrix metalloproteinases are able to degrade not only the basement membrane but also the extracellular matrix, allowing the exosomes to disseminate via the circulation. Exosomes are organotrophic, homing in to specific tissues such as bone. Here, they create the premetastatic niche devoid of cancer cells and cause an immunosuppressive environment, as well as induce changes in the host cells and produce myeloid-derived suppressor cells, of which some migrate to the primary tumour inhibiting the antitumour immune response further. Prostate cancer cells can disseminate even before the cancer is detected and thus escape curative therapy. If they survive the shear forces of the circulation and the antitumour immune response, they are able to implant in the premetastatic niche, transforming it into the metastatic niche. Here, they enter a latent state or dormancy period, which may last for months or years but later can “awake” to form metastasis. This review critically analyses the cellular and molecular mechanisms, which produce this process from cellular aspects to the signalling pathways responsible for this process. Multiple mechanisms are involved in a coordinated fashion to permit the survival of the cancer cells, from cellular changes in host cells and immunomodulation via chemokines and cytokines. It emphasizes the role of microRNAs and long non-coding RNAs in this process, and that patients with higher Gleason scores have a worse prognosis in terms of biochemical free survival at 10 years. Therefore, a precision medical approach may improve the biochemical free survival rate without affecting the role of the signalling pathways in normal cells. This includes the modulation of interleukin expression, elimination of exosomes, or the inhibition of important enzymes, such as MMP-2, thus mitigating the residual recurrence risk that persists with conventional therapy.

## 1. Introduction

In 2016, 19% of all American men diagnosed with cancer had prostate cancer, of whom 9% died [1]. By 2025, this figure had risen to 30% of all male cancers, a yearly increase of 3%, with an estimated prostate cancer death of 35,770 in 2025 [2]. Prostate cancer is heterogenous in nature, with different subtypes of prostate cancer cells existing in the same primary tumour. It has been reported that cancer cells can be detected in circulation even before a diagnosis is made [3,4]. Prostate cancer can also be divided into different subtypes with different phenotypic and genotypic characteristics, and this may affect treatment decisions [5,6,7]. It is important to note that prostate cancer cells do not exist alone but form a small part of the normal stromal microenvironment (SME).

It is important to note that prostate cancer cells do not exist alone but form a small part of the normal stromal microenvironment (SME). This micro-environment includes normal fibroblasts, which produce the essentials of the extracellular surroundings, including the secretion of collagen, fibrin, and proteoglycans. These aggregates form a matrix in the form of rigidity and tensional forces inhibiting the mobility of cells [8,9,10,11]. The SME also contains macrophages, CD4 FOXP4 regulatory T-lymphocytes, Natural Killer (NK cells), CD8 cytotoxic lymphocytes, B-lymphocytes, and regulatory B lymphocytes (Bregs). The SME also contains enzymes such as matrix metalloproteinase (MMP) 2 and 9, as well as cytokines and chemokines, which provide a dynamically changing microenvironment [12,13]. Therefore, the normal stromal environment is converted into an immunosuppressive tumour microenvironment by the cancer cells or via exosomes. The evidence is derived from in vitro experiments using prostate cancer cell lines: firstly, LNCaP cells, which have a mutated but functional androgen receptor. Secondly, PC-3 cells derived from bone metastasis, which are androgen independent and highly aggressive. Finally, DU145 cells derived from brain metastasis, which are also androgen independent but exhibit different characteristics as compared with PC-3 cells.

## 2. Material and Methods

To write this current mini-review information was carried out using a search of PUBMED. Most of the articles were published between 2019 and 2026. Only those articles prior to 2019 that were relevant to this review were included. Only those reviews and original articles published in English were considered. The key words used were “prostate cancer”, “tumour micro-environment”, “immunosuppression”, “exosomes”, “micro-metastasis”, “minimal residual disease”, “dormancy”, and “multi-omnics”. Those articles that were found to be duplicated were excluded, and full text documents were analyzed. Articles that lacked relevant or inadequate information were excluded.

## 3. Prostate Cancer Cells

Prostate cancer cells can transform normal fibroblasts into cancer-associated fibroblasts (CAFs). The interaction of prostate cancer cells and fibroblasts causes the proteolytic processing of basement membrane proteins, in addition to those caused by metalloproteinases [14,15]. In vitro experiments using normal prostate epithelial cells, fibroblasts, and different cancer lines may explain some of these molecular mechanisms. Using co-culture models, it has been reported that there is crosstalk between normal fibroblasts and normal prostatic epithelial cells and prostatic cancer cell lines [16]. This may be by direct cell-to-cell contact or via exosomes that are released by exocytosis into the SME [17].

**Figure 1:**
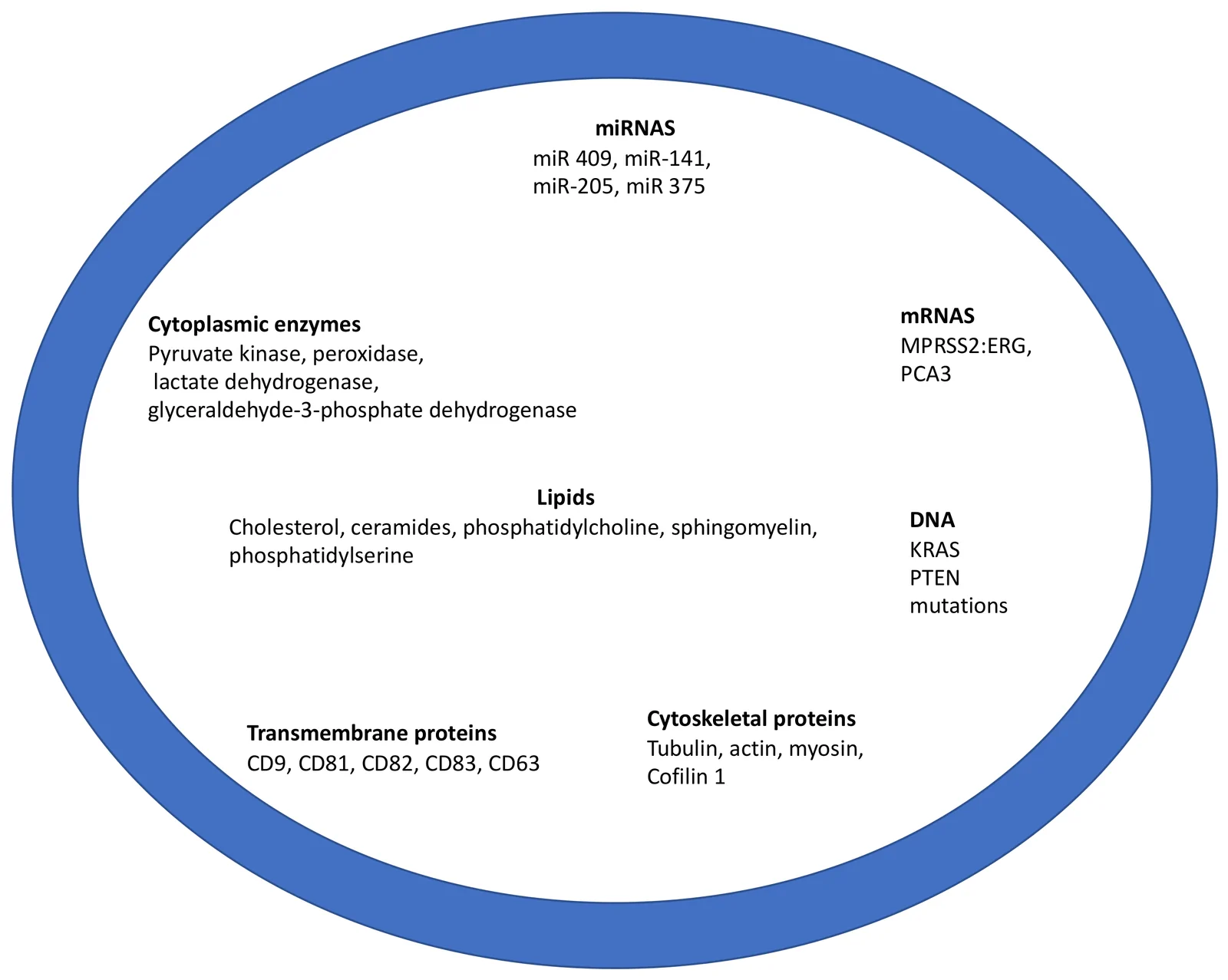
Schematic representation of Exosome heterogeneity.

Exosomes are nanoparticles 50–150 nm in diameter with a lipid membrane containing DNA, mRNA, microRNA, long non-coding RNA (ncRNA), enzymes such as matrix metalloproteinases 2 and 9 (MMP-2, MMP-9), cytokines, and chemokines [17]. Due to their lipid membrane, they can fuse with normal fibroblasts and release their contents, transforming them into CAFs. CAFs play an important role in re-shaping the primary tumour environment [17]. Different subtypes of exosomes are released by cancer cells and play an important part in inter-cellular communication by the transfer of their contents to other cells and thus can modulate the functions of the target cells [18] (Figure 1).

## 4. Exosomes

Exosomes play an integral part in causing immunosuppression in the TME and cancer cell dissemination [18].

Communication between cells in the TME of biological components has an important role in the maintenance of cellular homeostasis and the cells’ normal functions. However, in cancer patients, they play a detrimental function. The database Exocarta identified 9769 proteins, 3408 mRNAs, 2838 miRNAS, and 1116 lipids, suggesting they play an important role in intercellular communication [19]. Exosomes play an important role in the transformation of normal fibroblasts into CAFs in addition to increasing the immunosuppressive function of immune cells.

Exosomes and CAFs:

Exosomes, due to their lipid membrane, can fuse with normal fibroblasts, release their contents, and transform them into CAFs. CAFs increase the stiffening of the extracellular matrix, inhibiting the mobility and ingress of cells such as NK cells and CTLs [20,21], and direct migration to other tissues [19]. These morphomechanics of CAFs are associated with differing patient outcomes [22]. CAFs are different than normal stromal fibroblasts in terms of their triptosome expression [23]. As reported in in vitro experiments, there were differences in the expression of noncoding RNAs [18]. Using human prostate cells, obtained from benign pathologies or from cancer samples, the paraffin embedded samples were analyzed using immunohistochemistry and immunofluorescence staining. In the same study, male nude mice were injected subcutaneously with one of four different prostate cell lines. Using fibroblast activation protein (FAP), CAFs were identified. In prostate cancer specimens and stromal cells, YAP1 expression was significantly increased, promoting the transformation of normal fibroblasts into CAFs in vitro. This also increased the proliferation of cancer cells, increased cell migration, and inhibited apoptosis [24]. Similarly, CAFs increased the expression of the transcription factor SOX9.

**Figure 2:**
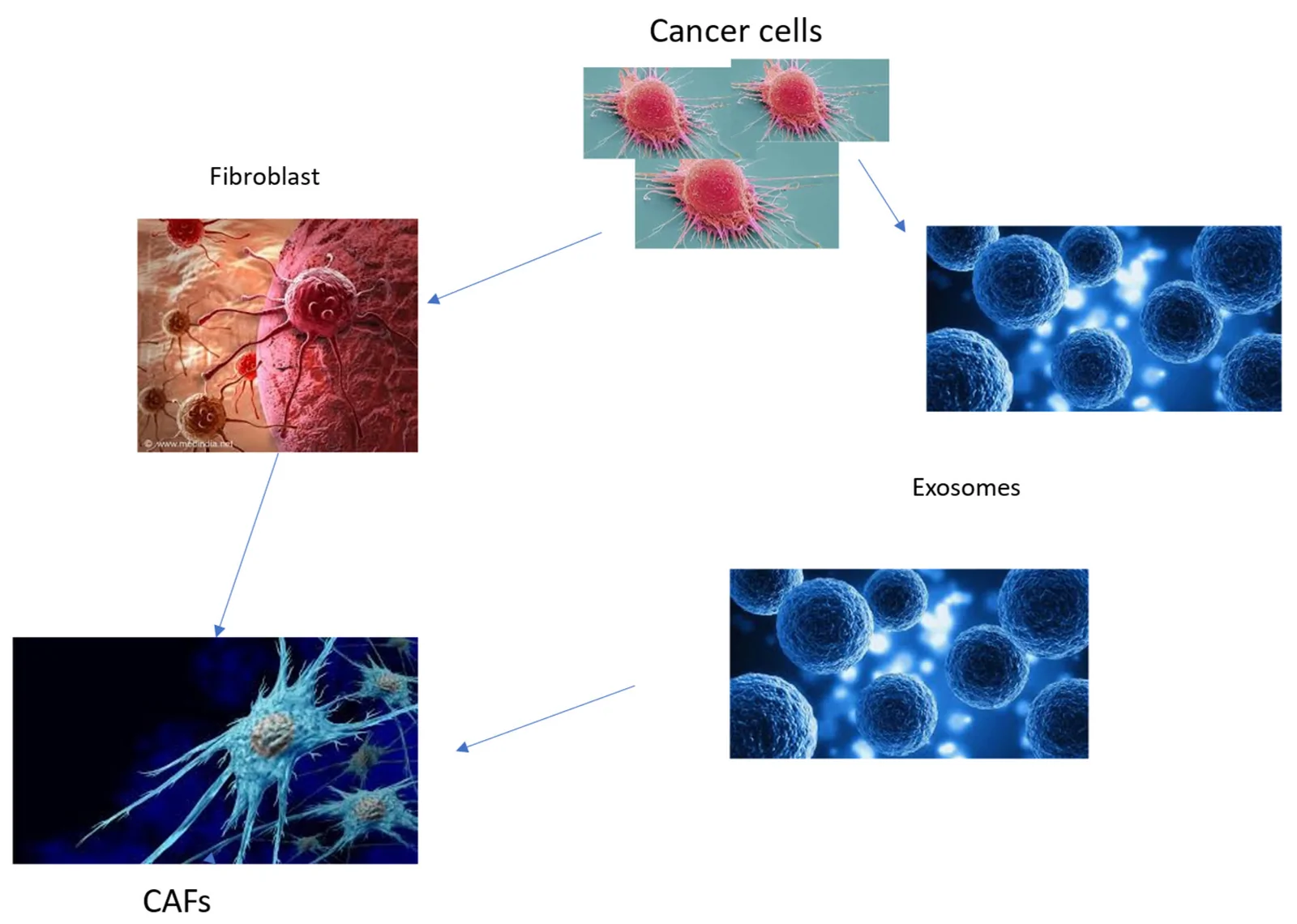
Schematic representation of the interactions between cancer cells and CAFS.

In vitro studies and in vivo experiments in mouse models showed that the proliferation and migration of cancer cells was increased via paracrine mechanisms [25]. Using co-culture organoids, it has been reported that CAFs can remodel the extra-cellular matrix (ECM) by increasing the expression of vimentin, fibronectin, and collagen, as well as increasing the invasive and migration potential of prostate cancer cells [26]. CAFs are heterogenous. There are two major subtypes using single-cell RNA sequencing from prostate specimens, with both being hormone-sensitive and hormone-resistant cancer cells [27]. CAFs-C1, but not CAFs-C0, was associated with hormone-resistant metastasis and a poorer prognosis [27]. This heterogenous CAF population, although lacking genetic mutations, is different from normal fibroblasts. There are methylation changes in the CAF DNA and modifications in the histone due to the increase of histone acetylation and decrease of histone methylation. As a result, CAF activation and cancer promotion are increased [28]. The authors also reported that miRNAs cause these epigenetic changes influencing gene expression, leading to a pro-tumour phenotype. In the TME, the increased collagen component inhibits the infiltration of the anti-tumour immune systems, limiting CTLs, increasing the recruitment of neutrophils, and downregulating the actions of interferons [29,30]. In organoid models, CAFs can upregulate the expression of ferroportin 1 and hephaestin, which causes an immunosuppressive TME by ferroptosis of NK-cells [31]. This occurs by recruitment of tumour-associated macrophages to the TME [31]. This is schematically represented in Figure 2.

There are differences between the transcriptosome expression of normal fibroblasts and CAFs. One study identified 818 differentially expressed mRNAs and 17 lc RNA and 15 potential ncRNA-mi (micro)-RNA-mRNA in CAFS. This may present a potential target for specific treatments [32]. The profile of CAFs is different between the primary tumour and metastasis [33]. In metastatic disease, the proportion of CAFs is increased, and there is also extra-cellular remodeling and TFG-β signaling. This is associated with a decreased survival of the patient. They also increased the infiltration by immunosuppressive immune cells and immunosupressive molecules [34]. CAFs are heterogeneous with differing properties. For example, C1 ABCA8 CAFs showed an increased proliferation as compared to other subtypes. They play an important role in the proliferation of prostate cancer cells, increasing the cancer cell growth and potential ability to cause metastatic spread [35]. Finally, in vitro experiments have reported the interaction between CAFs and prostate cancer cells changing the extracellular matrix and producing growth factors and matrix metalloproteinases, thus promoting cancer cell dissemination [35]. CAFs are also able to sculpt the TME by recruiting monocytes and inducing immunosuppressive PD-1^+^ TAMs [36].

## 5. Cancer-Associated Macrophages

The normal functions of macrophages are to phagocyose infective agents, dead or dying cells, and abnormal cells, including cancer cells. TAMs are derived from monocytes originating from the bone marrow. Infiltrating macrophages are known as cancer-associated macrophages (CAMs), with two different subtypes: M1 and M2. The M2 type promotes tumour progression by causing immune suppression, invasion, angiogenesis, and metastasis by remodelling the extracellular microenvironment [35,36]. CAMs form a distinct subtype of macrophages found in the TME, not only for their abundance but also for their plasticity [37]. After invading the TME, they differentiate into their mature forms, the so-called M1 and M2 phenotypes [38]. The TME is responsible for this differentiation through cellular signalling: M1 macrophages by interferon-gamma, while lipopolysaccharides and Toll-like receptor agonists play a role in inflammatory protumour immunity. Differing from this, M2 macrophages not only possess anti-inflammatory effects but also facilitate the growth of the tumour [39].

The M2 macrophages play an important role in the proliferation, metastasis, and immune evasion of the cancer cells by suppressing cytoxic T-lymphocytes (CTL) and natural killer (NK) cells [40,41]. CAMs produce antitumour mechansims. Some are suppressed and others are increased in terms of their suppressive antitumour effect [42]. Prostate cancer cells increase the recruitment of CAMs via the release of pro-inflammatory cytokines and chermokines [42]. Opsonins such as CD47 inhibit the phagocytosis of the tumour cells. CD47 is widely expressed on tumour cells, thus inhibiting the phagocytic capacity of macrophages [43]. This has been recently reviewed by Liu et al. [39,40]. TAMs are found mainly in areas of high CAF activity, implying intercellular crosstalk [44]. CAFs are able to recruit monocytes into the TME, while the precursors of macrophages cause their differentiation into immunosuppressive M2 macrophages, inhibiting the antitumour immune response [45]. M2 CAMs express an increased level of programmed death cell protein 1; this suppresses both the innate and acquired immune response, and it decreases their own anti-tumour effect [41]. There is positive feedback between CAMs and CAFs. CAMs induce the proliferation and invasion of CAFs [42]. As such, CAMs play a crucial role in the regulation between normal stromal cells, the TME, and cancer cells [42]. M1 macrophages can be distinguished from M2 macrophages via CD163, which is expressed only on M2 macrophages. Analysis of CAMs in human prostate cell lines using mouse models.

Reported that, in the TME, at least 94% of CAMs showed an M2 phenotype [46]. Using the supernatant obtained from PC-3 prostate cell lines in mouse models, it has been reported that the profile of macrophages increases in the direction of the M2 phenotype [47,48]. Changes in the metabolic patterns and the creation of a hypoxic environment is a key feature of solid tumours. By modulating the antitumour immune response, it promotes proliferation, invasion, and metastatis in prostate cancer [49].

Using in vitro models, M1 macrophages can secrete IL-4, Il-10, and IL-13, which induce pro-tumorgenic and proangiogenetic M2-like features [42]. The histological localization of CAMs found in hypoxic and/or necrotic cancer niches is associated with a worse prognosis [50]. Prostate cancer cell microarrays use tissue obtained from prostate cancer and adjacent normal tissue. Imaging mass cytometry and an analysis using HIF-1α (a marker of hypoxia) and CD68 are used to identify macrophages, YY1, a transcription factor, and DNA1, a nuclear marker, in the hypoxic regions. YY^+^ macrophages were found to be significantly higher than YY^−^ macrophages [51].

Furthermore, these macrophages were found to have an upregulation of 193 genes as determined by RNA-sequence analysis [51]. Using in vitro and in vivo models, it was found that TAM-derived exosomes containing miR-95 are partly responsible for cancer progression and are associated with worse clinicopathological features [52]. Exosomes enhance M2 polarization through different mechanisms; the exosomal RNF157 mRNA secreted by prostate cancer cells increases M2 macrophages by destabilizing HDAC1 [53], similarly using PC3 prostate cancer lines. The transfer of miRNA Let-7B induces the conversion of normal macrophages into CAMs. Although LET-7B is expressed significantly higher in non-cancerous cells as compared to tumour cells, in exosomes, the reverse is true, implying that the expression of LET-7B is associated with prostate cancer progression through the action of CAMs [54].

Exosomes produced by prostate cells also inhibit their phagocytosis by macrophages via EIF3B-mediated exosomal sorting of miR-100-5p. In vitro experiments used normal prostate cells and PC3 cell lines, showing that the transfer via EIF3B increases its expression in macrophages. The eukaryotic translocation initiation factor 3 subunit B is highly expressed in prostate PC3 cell lines and bound specifically to miR-100-5p, reducing phagocytosis by macrophages [55]. Thus, CAMs play a multifunctional role in the creation of an immunosuppressive TME.

## 6. CD4-FOXP3 Regulatory T Cells (Tregs)

Prostate cancer cells are able to recruit CD4-FOXP3-regulatory T-cells (Tregs) into the TME, as well as CD8^+^ CTLs. The key regulators in this process are macrophages in recruiting both types of lymphocytes via the CXCL12/CXCR4-signalling pathway. Tregs directly alter the CTL function, and Interlukin 2 is critical in reshaping the TME. Tregs deplete interleukin 2 and, via interleukin 2/STAT5 pathways, cause CD8^+^ CTL exhaustion, thus increasing the immunosuppressive nature of the TME. Cancer cells also cause the proliferation of Tregs, further increasing immunosuppression of the TME. CD4^+^ T lymphocytes subtypes are diverse, with each subset having its own function. For example, T-cells of the Th1 subtype are associated with a better prognosis and outcome [56]. CD8^+^ T-lymphocytes are cytotoxic and associated with a better prognosis. There is a correlation between the number of CD8^+^ cytotoxic T-cells and an improvement in disease-free survival [56,57]. This differs from Tregs, as the major regulation of its function is the transcription factor FXP3, resulting in a worse prognosis and more advanced stages of prostate cancer [58,59]. The C-C chemokine receptor 4 (CCR4) is expressed in Tregs and has an important role in the infiltration of Tregs into the TME [60]. This is associated with a worse prognosis and an increased progression to castrate-resistant prostate cancer [61].

## 7. B-Cell Lymphocytes and Regulatory B-Cell Lymphocytes (Bregs)

B lymphocytes cancer either inhibits or promotes the progression of prostate cancer via the production of antibodies against specific antigens. These antibodies may directly suppress tumour proliferation, which inhibits the proliferation of tumour cells and increases T lymphocyte responses. This favours immune attacks on the cancer cells vis6inhibit3. The counterpart of B-lymphocytes is Bregs, which produces immunosuppressive cytokines, IL-10, IL-35, and TGF-β and thus is able to maintain an inflammatory response and an immune response [62,63]. It is able to inhibit and promote immunosuppression via direct interactions with Tregs, macrophages, and NK cells, thus increasing the immunosuppressive TME [63,64,65,66,67]. In gynecological cancers T cells and granzyme B, Bregs is found to be adjacent to one another. Therefore, this may possibly the cause immune tolerance and permit the survival of cancer cells [68]. However, research on the role of Bregs and its role as a critical humoral immune response is poorly understood. This is especially so in terms of systematic, phenotypic, and functional analyses [69,70]. An analysis using multi-omics based on Breg-related genes showed that a high expression of the gene SIVA1 was significantly associated with a shorter biochemical failure-free survival, and immunohistochemical and western blot analysis confirmed an association between SIVA1 and the immune-checkpoint molecule Lymphocyte Activation gene 3 [71]. Evidence of the role of cancer-infiltrating B cells and plasma cells shows they have an important and multifaceted role in the antitumour tumour response in patients with prostate cancer [72,73]. SIVA1 is a transcriptional target of p53, promoting ATS inactivation via Hdm2, resulting in the inhibition of p53-mediated apotosis and an E3 ubiquitin ligase for p14ARF, thus indirectly increasing p53 inactivation [74]. SIVA1 also increases T cell apoptosis by negatively regulating the nuclear factor NFkappaB-signalling pathway, promoting T cell apoptosis [75]. By regulating microtubule dynamics, it is able to inhibit cancer cell migration and the epithelial–mesenchymal transition, thus decreasing cancer cell migration from the primary tumour [76]. The relation between SIVA1 and LAG3 remains to be confirmed, as in a direct mechanistic link with Bregs. This study was conducted using PCa cell lines and not immune cells, thereby not confirming a direct cell-immune mechanism [76].

## 8. Antigen-Presenting Dendritic Cells

Dendritic cells (DCs) form an integral part of the immune system; mature DCs show an increased level of the Class II major histocompatability complex (MHC), as well as increased levels of surface CD80 and CD86. It acts as a costimulate to MHC II [77,78]. DCs play an important role in the activation of both CD4 helper T-cells and CD8 cytotoxic T-cells, where direct cell-to-cell contact is required for this process [79]. Exogenous peptides are internalized into the DCs, and then, in combination with MHC II molecules, they increase the activation of CD4^+^ T-cells. As a result, this increases the activation of other immune components such as CD8^+^ cytotoxic T-cells, macrophages, and B-cells. The interaction between DCs and B cells causes isotype switching, increasing the diversity and specificity of the produced antibodies [80].

However, the TME is able to adapt, causing immature DCs to be dysfunctional. Thus, the tumour antigen presentation of DCs is defective. In these tumour-infiltrating dendritic cells (TIDCs), the co-stimulatory molecule expression is decreased; this impairs the activation of CD4^+^ and CD8^+^ T cells [81]. This is another mechanism by which tumour cells cause an immunosuppressive TME. New analytic methods such as single-cell RNA sequencing has made it possible to identify and characterize rare and new cell types [82], as well as cell-to-cell communication via ligand receptor signaling [83]. However, studies on this type of immune evasion have been limited, and thus the complete understanding of the TME immune-evasion system has yet to be achieved. Feriz et al. [80] reported that using single-cell RNA sequencing of TIDCs in prostate cancer specimens showed a higher number of them in the TME than in normal tissue. Their study showed that some signalling pathways, such as TNF-α/NF-κB, had an increased activity in TIDCs as compared to healthy prostate cells. TIDCs are able to secrete an increased quantity of chemokines, cytokines, and growth factors as compared to normal DCs [84,85]. These results were consistent to these reported by Wang et al. [81], and this differential expression of TNFα in prostate cancer increases the immunosuppression of the TME [86]. These results were contrary to earlier reports. However, the secretion of chemokines and cytokines plays an important role in the survival, proliferation and dissemination of prostate cancer cells, as well as the integration of immune suppressor cells into the TME.

## 9. Myeloid-Derived Suppressor Cells (MDSCs)

MDSCs are derived from bone marrow monocytes found in the pre-metastatic niche of the bone marrow. The pre-metastatic niche is an immunosuppressive zone of the bone marrow caused by the infiltration of exosomes (see later). Two distinct subtypes of MDSCs have been described: those that are similar to immature granulocytes (G-MDSC), and those that are similar to monocytes (M-MDSC) [87]. Each has different effects on the immune system, such as T cell inhibition [88], inhibition of DC maturation [89], energy of NK cells [90], and the expansion of Tregs in the TME [91]. Chemokines released from TME cancer cells can cause differentiation of MDSCs into epithelial cells, fibroblasts, and CAMS [92], as well as osteoblasts found in the pre-metastatic niche [93].

MDSCs that become activated cause an immunosuppressive and pro-tumourigenic TME through cell-to-cell contact and, secondly, via cytokines. The use of an in vivo model for PTEN-negative mice reported an increase in MDSCs as compared to normal wild-type mice, and the difference increased with disease progression [94]. Comparing G-MDSCs with M-MDSCs, the first type has increased numbers in metastatic sites as compared to the primary site [95], whereas M-MDSCs were more frequently found in the circulation and associated with resistance to prostate cancer therapies [96,97,98]. A meta-analysis concluded that elevated levels of circulating MDSCs were associated with a worse overall survival as compared with those patients with low MDSC levels [99]. The expression of PD-1 in exosomes produced from prostate cancer cells increased the MDSCs’ abililty to increase tumor immune evasion, as did mitochondrial DNA released from exosomes [100].

**Figure 3:**
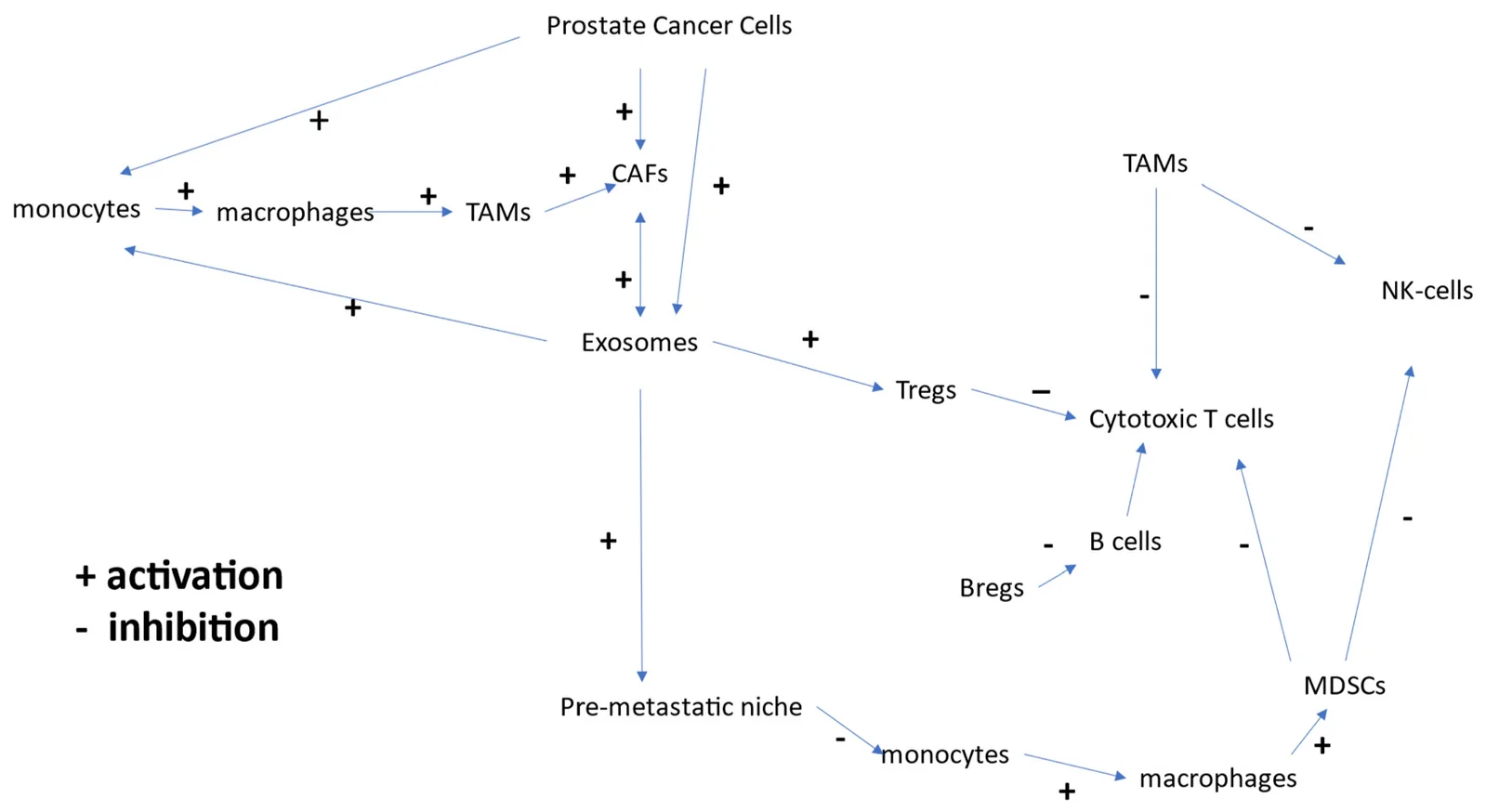
**Schematic representation of cell-to-cell interactions:** MDSCs migrate to the primary tumour via the CXCR4-signalling pathway, although the full mechanism of this is unclear.

However, there are limitations in translating from animal models to humans, especially because of differences between the murine and human immune systems and how to define a cut-off point and the methods of analysis of the results. A lack of standardization impedes comparisons between studies, as does the methodology of analysing MDSCs [101,102]. In hypoxic conditions, MDSCs have a survival advantage showing increased differentiation and functional activity, an increased infiltration from the pre-metastatic niche to the primary tumour site, and the remodelling of the TME. The increased functional activities inhibit both CD8^+^ cytotoxic lymphocytes and NK cells [103]. Figure 3 shows a schematic view of the cell-to-cell interactions.

## 10. Hypoxic-Driven Immunosuppression in the Tumour Microenvironment

Hypoxia in the TME increases its immunosuppressive effect while cancer cells continue to proliferate [104]. This hypoxic state has been well established; cancer cells switch their metabolism through the glycolytic pathway. Although it produces less ATP from each glucose molecule, the faster kinetics, as compared with the tricarboxylic acid cycle, produce no net difference in ATP production [105]. This metabolic change produces an increased production of lactate, causing an acidic TME. This change to an acidic TME causes a change in the composition of the TME stroma and ultimately increases the potential of cancer cell dissemination. In hypoxic conditions, all of the immune cells are affected. The maturation of DCs is unaffected in a hypoxic environment but causes dysfunctional DCs. Antibody uptake is inhibited, the expression of cytokines changes, and there is downregulation of differentiation and activation markers such as CD40, CD80, and MHC II [106].

Macrophages are polarized into the M2 phenotype, with there being an increased infiltration in hypoxic or necrotic areas of the TME [107]. B-Cells are also affected. It has been well established that hypoxia-mediated channels, especially HIF-1α, demonstrate an increased infiltration in those areas of the TME, although this has not been well characterized or fully understood. HIF-1α increases glycolysis in the B cells, allowing them to proliferate in the hypoxic TME [108]. HIF-1α is also important in the function of T-cells. Its activation promotes the expression of glycolytic genes and post-translational modifications. Activated T cells need increased energy to produce macromolecules, intracellular mediators, and cytokines. This is achieved by an increase in glucose and glutamine catabolism for the synthesis of nucleotides and lipids, while oxidative phosphorylation continues forming ATP [109]. A change in the metabolism of glucose shifts the differentiation of naïve T cells to Tregs. In the hypoxic TME, the number of cytotoxic lymphocytes is decreased and has an impaired function due to high lactate levels [110].

**Figure 4:**
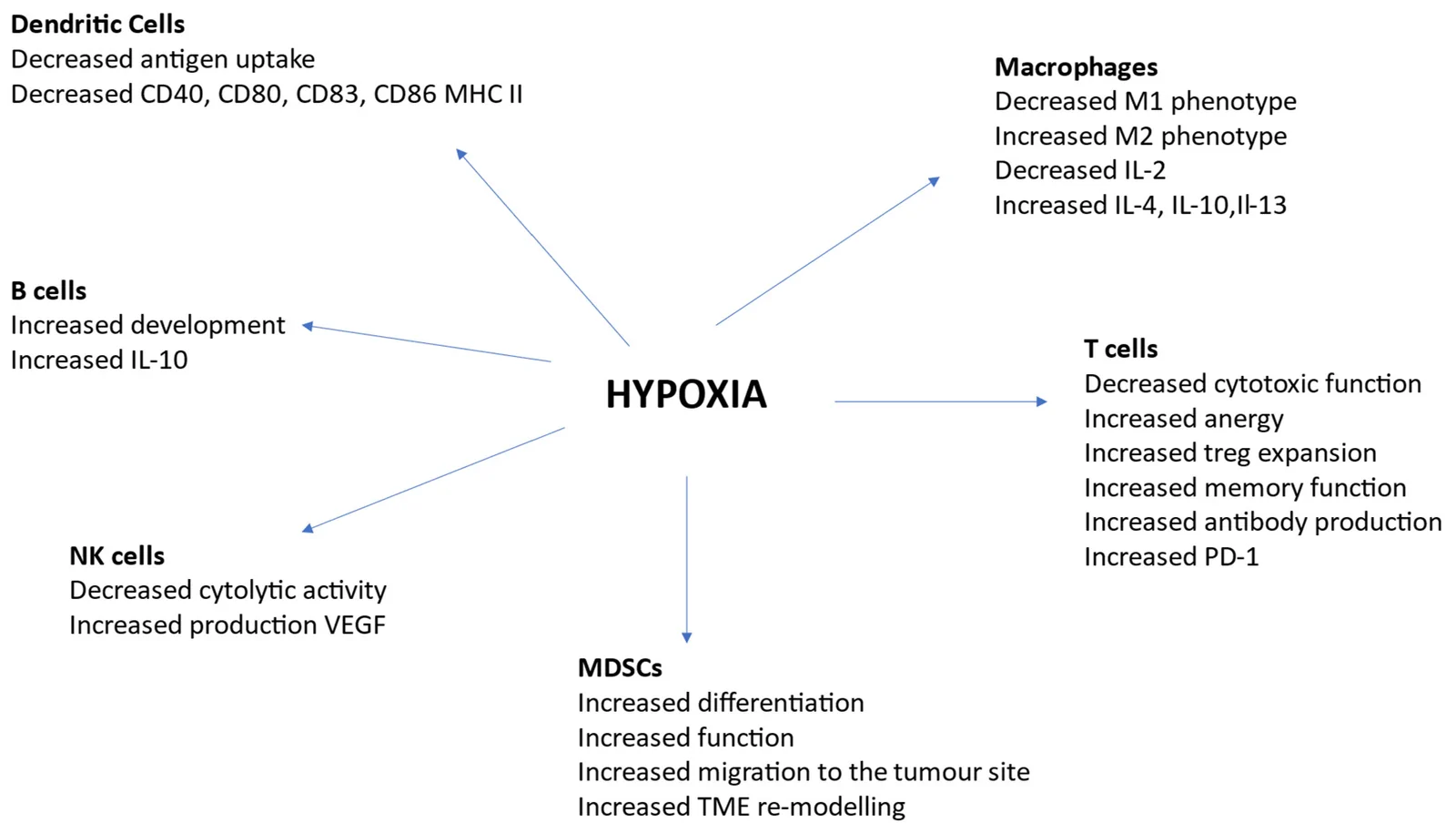
**The** **effects of hypoxia on the immune system.**

NK cells are also affected with their cytotoxic activity by downregulating the expression and function of NK receptors [111]. These changes caused by hypoxia have been extensively reviewed recently by Vito et al. [46]. These changes in the metabolism of the immune system favour tumour progression. Using in vitro models in different cancer cell lines, it has been reported that using RNA sequencing in in vitro, in vivo mouse models and human hepatocellular carcinoma cells, whereby macrophages were stimulated using unsaturated fatty acids, promoted cancer proliferation by FAMP5 activation, which resulted in the inhibition of antitumour T cell activity [112]. The authors concluded that FABP5-mediated unsaturated fatty metabolism in CAMS is important in the modulation of antitumour T-cell functions. Furthermore, the TME is changed by arginine metabolism of the cancer cells, although how this interaction with other cellular components functions or by what mechanisms remains unknown. Using in vitro breast cancer cell lines, the production of arginine induced a M2 polarization of CAMs and, as a consequence, inhibited CD8^+^ cytotoxic T cells. Normally, there is increased T cell activation as a result of arginine metabolism, but this was overridden by the CAM–cancer cell interaction [113]. Using in vitro human prostate cancer cells and single-cell RNA sequencing lead to the identification of a subset of CAMs, which showed a dysregulation of the lipid metabolic transcriptional pathways. The presence of these lipid-loaded CAMs was associated with cancer progression and a shorter disease-free survival. The accumulation of lipids in CAMs is dependent on Marco, whose expression is enhanced by cancer IL-1β secretion. This leads to increased cancer cell dissemination via CCL6, secreted by these lipid-loaded CAMs [114,115]. This is schematically represented in Figure 4.

Matrix metalloproteinase-2 (MMP-2) is a gelatinase and has an important role in prostate cancer. It is able to degrade the basement membrane, and the extracellular matrix releases immunosuppressive cytokines and chemokines. It is produced as an inactive zymogen and activated by other enzymes such as MMP-1 or free radicals via the cysteine switch [116,117]. By degrading the extracellular matrix, it is able to create pathways in order for exosomes and, later, cancer cells to disseminate into the circulation, with the cancer cells being termed circulating tumour cells (CTCs). MMP-2 is also able to increase the suppressive immune system. We predicted biochemical failure in men with bone marrow micrometastasis after curative therapy [118]. The role of MMP-2 in the metastatic cascade has recently been reviewed [119].

## 11. T Cell Exhaustion

CD8^+^ cytotoxic lymphocytes are a crucial element of the antitumour immune response; they need to be in direct cell-to-cell contact with cancer cells to be effective. They are present in the initial TME. However, with time, their functional capabilities decline. However, the understanding of the mechanisms that cause a decrease in their functional capabilities in the prostate cancer TME remains largely unknown [120]. It has been suggested that the continuous stimulation of cytotoxic T cells by cancer cell antigens results in the gradual expression of an increasing number of immunosuppressive receptors, along with a decrease in the release of antitumour cytokines. This combination leads to a decline in their antitumour role and is defined as T cell exhaustion [121,122]. These T cells are distributed within the TME, and their functional decline allows the prostate cancer cells to escape their cytotoxic role and thus increases the immunosuppressive environment. It has been suggested that this functional change may be one important mechanism in the limited success of immunotherapy in prostate cancer patients [123,124]. Multiple mechanisms causing this effect have been suggested by direct targeting of T cells by cancer cells or the secretion of soluble factors [124]. These soluble factors include various cytokines such as IL-2, TGF-β, and IL-14 [125,126,127]. Others include the PD1/PD-L1 inhibitory receptor signaling, TIM-3, and the signaling pathway PI3K/Akt/mTOR [128,129,130]. Long-term exposure to TME metabolic products has been another suggested mechanism [131].

It has been reported that there is a possible association between prostate cancer exosomes and CD8^+^ T cells, although the mechanisms to achieve this remain unclear. Using exosomes derived from prostate cancer lines, Salimu et al. [127] reported that CD73 expression inhibited the cancer antigen presentation to DCs, and, as a consequence, inhibited T cell function [132]. Using DU145 prostate cancer cells, the authors transduced them with shRNA, which knocks down Rab27a and, as a consequence, inhibits the secretion of exosomes. For the resulting cells, there was a significantly higher antigen-specific T cell response when combined with DCs. The addition of purified exogenous DU145 cells prevented the T-cell response, implying that the dominant effect of the exosomes was not antigen presentation but immunosuppression.

Exosomes secreted by cancer cells alter the function of myeloid cell function through the inhibition of monocyte differentiation of DCs to promote immunosuppressive myeloid cells. Exosomes isolated from the plasma of patients with advanced melanoma revealed a similar effect on monocytes, causing TGF-β-mediated T cell function inhibition [132]. Using cultured mouse mesenchymal stem cells (MSCs), it has been reported that the supernatant has immunosuppressive effects on immune cells, including T cells via apoptosis and cell-cycle arrest. MSC-derived exosomes significantly suppressed T cell function by causing cell-cycle arrest [133].

Using in vitro studies with PC3 prostate cancer cell lines and in vivo mouse models, it was reported that CD8^+^ T cells could uptake exosomes, up to 85% after 48 h of incubation, producing T cell exhaustion. The authors reported that inhibitory PD-1, TIM-3 IL-10, and TGF-β were increased, while IL-2, Il-4, IFN-γ, and TNF-α effector cytokines were decreased [134]. Thus, it can be seen that the cause of T cell exhaustion is multifactorial, but it is associated with prostate cancer progression.

## 12. Exosomes

Exosomes play a pivotal role both within the primary TMA, systemic spread, organotrophy, and the formation of the pre-metastatic niche. They are nanoparticles of between 50–150 nm with a lipid membrane. They contain DNA, mRNA, miRNA, lnc-RNA, and enzymes, including MMP-2, chemokines, cytokines proteins, and lipids [135,136,137]. Cancer cells release different subtypes of exosomes, especially with regards to their composition and interactions between cancer cells and CAFs [137]. Immune cells may uptake exosomes, which results in immune suppression, transferring their contents into monocytes, altering the expression of CCR6 and CD44v7/8, which activates the Akt pathway, resulting in the conversion of the normal monocytes into CAMS [137]. They also increase the immune-suppressive environment by activating Tregs via TGF ß-1 [137]. Exosomes not only play a part in oncology but form part of normal physiological processes [138]. In patients with prostate cancer, they are released from cancer cells by exocytosis and by having a lipid-based membrane, which easily can enter normal host cells, where they release their contents and regulate the TME [139].

They remodel the gene expression and the molecular structure of the premetastatic niche. The cell-adhesion receptor integrins regulate organotrophic homing of cancer and immune cells and circulating exosomes [140,141]. The integration of these exosomes modulates the host defence mechanisms, as well as anti-tumour lymphocytes [139]. Exosomes also home into specific tissues via cytokines, derived from the TME, by binding to cells that express the specific cytokine receptor and thus their biodistribution [142]. Exosomes are regulated by autocrine and/or paracrine mechanisms, and the TME aids their release [135,136,137].

HIF-1 increases the production of exosomes significantly in a time-dependent manner, secreting and activating Rab22A. The overexpression of Rab22A effects the morphology and function of early exosomes and alters the recruitment of exosomal proteins. Upregulating Rab27A and decreasing the expression of Rab7, LAMP1/2, and Neu1 increases exosome dissemination from the cancer cells, as does the activation of TGF-α/Akt/PRAS40 [135,136,137]. As previously mentioned, exosomes express intergrin β1, which is associated with bone as a metastatic target tissue [135]. Phosphorylation of Talin 1 via Cdk5 kinase activates β1 integrin, allowing binding to the normal cells in the target tissue [140]. The process has been recently reviewed by Geng et al. [137]. The exosomes enter the host cells and release their contents, causing various changes in the normal microenvironment [141].

They cause immunosuppression, with a decrease in the number and anti-tumour immune response in CD4, CD8, and NK-cells [143]. Exosomes also inhibit CTL production via the expression of PD-L1, reducing their capability of proliferation, production of cytokines, and thus anti-tumour response [143]. They are also capable of activating TGFß, causing the activation of the TGFß/SMAD-signalling pathway in normal fibroblasts, converting them into CAFs [144]. The exosomes are able to firstly initiate the epithelial to mesenchymal transition, thereby facilitating cancer cell dissemination. Secondly, they prepare the pre-metastatic niche, which is without cancer cells present, and transform the host cells to change the normal environment to be immunosuppressive in nature [145]. miRNAs are RNAs that are noncoding and play a part in the epithelial-to-mesenchymal transition, allowing cancer cells to disseminate. They are able to change the normal stromal microenvironment into a TME, while the expression of miRNA via DLK-DIO3 stem cells is associated with treatment failure [48,96]. miRNAs therefore have an important role in the premetastatic niche. They are able to change bone remodelling and help support cancer growth. The expression of miRNAs enables them to bind to other miRNAs, affecting osteoblast and osteoclast differentiation and the proliferation of the tumour cells by abnormal remodelling of the TME, thus enabling tumour growth [146]. Under the relative hypoxic conditions of the bone marrow, MMP-2 activity is increased. This is increased by an increase in the production of MMP-9, as well as fibronectin, collagen, and CD11b-positive cells. Under hypoxic conditions, MMPs remodel the TME to a protumour TME [147]. The role of PD-L1 is to increase the elimination of cancer cells, and it is expressed on the surface of CLTs. However, cancer cells also express variable quantities of this protein. This is correlated with aggressive prostate cancer, while those with high Gleason scores are correlated in a negative form of CTL infiltration. The secretion of PD-L1 in the premetastatic niche is increased by the JAK/STAt3 pathway by increasing the number of myeloid-derived suppressor cells (MSDCs). These are able to suppress CTL infiltration and thus increase the immunosuppressive environment, decreasing the anti-tumour immune response [148]. MDSCs also promote cancer progression by miR-95 transfer to cancer cells. In the premetastatic niche, the levels of miR95 are increased, possibly by binding to cancer cells and as a tumour promotor to JunB, thus increasing the proliferation, invasion, and the epithelial to mesenchymal transition. This results in the higher expression of this biomarker and is associated with a worse prognosis, linking the miRNA-95/Jun-b-signalling pathway as a possible target for treatment [149]. MDSC cells also increase the formation of the premetastic niche by immunosuppression, vascular leakage, remodelling of the extracellular matrix, and angiogenesis. They also, by non-immunological pathways, promote cancer proliferation and metastasis [150,151]. In addition to remodelling, the TME is also able to disseminate NK-cells and further inhibit CTLs, increasing the immunosuppressive environment of the primary tumour [152]. This was recently reviewed by Xu et al. [148]. The miRNAs are associated with the progression of prostate cancer. Elevated levels of miR-19a-3p and miRNA 23b-3p are found in aggressive cancer as compared to low-grade tumours [153].

There is an association between miR-7-5p with the number of CAMs. mRNA-96 increases cell-to-cell communication through upregulation of the adhesion molecules E-cadherin and Epcam, which increases the tumour cells’ ability to bind to osteoblasts and, as such, increases the proliferation of tumour cells [154].

Furthermore, microvesicules also increase cancer cell proliferation and integrate with adipocytes, fibroblasts, and mesenchymal stem cells [155]. Overexpression of CXCR-4 is promoted by TFG-β, with an elevated expression of CXCR-4. There is also an increased potential for cancer cells to migrate. SDF-1, in the microenvironment, increases the ability of CXCR-4 cancer cells to invade target tissues [156]. Studies have reported that SFF-1 expression is associated with increased CXC.E.4 expression, inducing the growth, proliferation, and migration from the premetastatic niche [156]. ST6 β-galactoside α2,6-sialltransferase (ST6GAL 1) is upregulated in prostate cancer. It mediates α2-6-linked sialylation of N-glycosylated proteins and is released from exosomes. It exists as both a soluble and membrane form, and it is associated with aggressive prostate cancer [157]. ST6GAL1 is transferred in its soluble form to the cancer cells due to the expression of Nogo-66. Receptor homolog 2 (NgR2) and β3 intrigrin inhibit this transfer and are increased in aggressive prostate cancer and may promote the risk of metastasis in part by the same mechanisms that form the premetastatic niche [157].

MMP-2 does not only create pathways for the dissemination of exosomes but later tumour cells, where they are known as circulating tumour cells (CTCs). These cells home into the premetastatic niche; they may also implant in other tissues but do not proliferate. Exosomes are also found under hypoxia conditions in the bone marrow and are able increase the activity of MMP-2 in the premetastatic niche [147]. Exosomes also transform bone marrow monocytes and macrophages into myeloid-derived suppressive cells, which, as mentioned previously, inhibit the antitumour immune response. Some disseminate to the primary tumour, while others remain in the bone marrow premetastatic niche. MDSCs are immature macrophages; myeloid-derived and dendritic cells are related to a poorer prognosis and increased immunosuppression in the TME [158]. They have been detected outside of the blood stream, including the primary tumour, as well as pre-metastatic and metastatic niches [159]. In these sites, the number of MDSCs increases, as well as their differentiation. These processes are mediated by a variety of molecules and growth factors. These include granulocyte–monocyte, granulocyte, granulocyte–macrophage growth factors, and the vascular endothelial growth factor [160]. This transformation to MDSCs is caused by primary tumour exosomes through miRNAs and non-coding RNAs, and miR-494 induces the activity of TGFβ [161]. An increased level of IL-6 has been associated with an increased immunosuppressive TME by increasing the number and activity of MDSCs [162].

Recent studies have suggested the use of urinary exosomes for the early detection of prostate cancer rather than the use of PSA. Wei et al. [159] studied the use of urinary exosomal PSA (UE-PSA), taking urine samples prior to a prostate biopsy. Using an ELISA method, they reported that UE-PSA obtained an AUC of 0.953 to distinguish prostate cancer from a benign disease and an AUC of 0.879 between non-significant and clinically significant prostate cancer. Urine analysis is non-invasive, while the analysis of DNA, RNA, and proteins contained within the exosomes can become diagnostic markers. An aggressive prostate cancer phenotype is predicted by the detection of ERG, PCA3, and SPDEF mRNAs. There is down-regulation of miRNAs such as miRNA-196, miRNA-501, and miRNA-34, and up-regulation of miR-574-3p, miR 141-5p, and miR 21-5p. Artificial intelligence combined with machine learning has meant the development of smart platforms for more precise detection and earlier diagnosis of prostate cancer [163,164].

New techniques provide more sensitive detection and characterization of urinary exosomes, such as surface plasma resonance sensors, flow cytometry, and digital-droplet PCR. However, there remains the problem of exosome heterogeneity, their origin, size, and molecular variation. A lack of a standardized method of isolation and the storage of the exosomes await analysis [165,166,167].

## 13. The Role of the CXCR4/CXCL12 and TNF-β in the TME

Li et al. [164,168], using tissue dissociation obtained from healthy prostate tissues, adjacent normal prostate tissue, and localized prostate cancer combined with scRNA datasets, studied the CXCL12-CXCR4 axis in the TME. They reported that there was communication between different cell types in the TME. Using differential expression analysis (DEA), they investigated the relationship of the CXCL12-CXCR4 ligand receptor (L-R) and different immune cells within the TME. The interaction between macrophages, cytotoxic CD8^+^ T cells, Tregs, and tissue-resident T cells was significantly increased. This underlined the importance of CXCL12 produced by macrophages in recruiting Tregs and CD8 cytotoxic T cells from the circulation and lymph nodes into the TME. Tregs can deplete and sequester IL-2, which causes the inhibition of the activation and cytotoxicity of CD8^+^ T cells [169]. It was suggested that the sequester of IL-2 by Tregs leads to the exhaustion of cytotoxic T cells in the juxta-TME. This exhaustion of cytotoxic T cells was mediated through IL-2/STAT5 signalling, which upregulated the thymocyte selection-associated High Mobility Group Box Protein (TOX) [170]. Therefore, this indicated that the CXCL12-CXCR4 LR-signaling axis is able to recruit Tregs, as well as cytotoxic CD8 T-cells, but it causes functional exhaustion of the cytotoxic T cells [168]. Using single-cell and spatial transcriptomic profiling from healthy prostate tissue and the differing subtypes of prostate cancer, including the neuroendocrine subtype, it was reported that the TME undergoes a coordinated reprogramming during tumour progression and after neoadjuvant androgen blockade. This causes distinct cellular responses; TGF-β is upregulated by CAMs to establish immunosuppressive niches, while CXCL12-positive CAFs maintain the spatial co-localization and facilitate immune cell recruitment [171]. Especially in specific CAM subsets, this enhanced chemokine signaling, as revealed by spatial analysis, increased cytotoxic T cell exhaustion and Treg proliferation, thus connecting epithelial plasticity with reprogramming of the TME [171].

As mentioned earlier, the CXCL12-CXCR4 chemokine-signaling axis as one part of its multifunctional role is immune cell trafficking. Prostate cancer cell-derived exosomes, which are able to upregulate CXCR4 via the TRL2/NF-κB-signaling pathway, can recruit MDSCs into the TME. Li et al. [168], using mouse spleen and tumor tissue, showed that MDSCs were significantly increased in these tissues when exosomes were injected into the mouse tail vein and detected using flow cytometry. They also showed using the in vitro transwell chemotaxis assay that MDSC recruitment was enhanced by exosome stimulation, while a significant increase in the levels of MDSC CXCR4 was shown using western blot and flow cytometry. The authors concluded that the TLR2/NF-κB-signalling pathway promoted MDSC migration into the TME via the CXCR4-CXCL12 pathway [172]. This could be one explanation of how MDSCs migrate from the premetastatic niche to the primary tumour as a result of exosome infiltration into the premetastatic niche [173].

However, the interpretation of the results must be taken with caution. In vitro studies using PC3 prostate cancer cells determined that by using gene-expression data and flow cytometry, PC3 cells expressed low levels of CXCR4 transcript levels but a higher proportion of CXCR4 positive cells as determined by flow cytometry. As such, transcriptional activity does not necessarily signify a high surface protein availability. This has been suggested to be due to active post-translational regulation, such as rapid receptor internalization. Therefore, it is important to validate both the gene expression level and protein expression in different prostate cancer cell lines prior to therapeutic studies [173]. Furthermore, expression patterns may vary according to the passage number, culture conditions, and genetic drift, which shows that validation of the results is crucial and that different methods may result in different results and conclusions [173].

## 14. The Role of TGF-β1 in Prostate Cancer

TGF-β1 is a part of the TGF cytokine family, playing a role in a number of cellular functions. It can act as an immunosuppressive molecule by maintaining the phosphorylation of the retinoblastoma protein and the induction of p15 and p21 transciption, and by inhibiting c-Myc cancer growth signalling [174]. In terms of its function in cancer patients, it has the capacity to promote tumour proliferation and recruit immunosuppressor cells into the TME [175].

In prostate cancer, TGFβ is overproduced, partly from its secretion by CAFs and CAMs. The cytokine induces the transformation of CAFs in an independent fashion. Together with the action of EGF, it potentially can induce the growth of other fibroblast cell lines by anchorage independent growth. In response to TGF-β stimulation, CAFs produce mitogenic signals, resulting in the promotion of tumour survival and proliferation [175]. TGF-β has an important role in the process of immunosuppression, angiogenesis, and formation of metastasis [176]. Tregs also secretes TGF-β to directly impair CD8^+^ cytotoxic T-cells, thus improving tumour cell-immune evasion [177,178].

TGF-β is not only produced by, but also regulates, immune cell function, such as macrophages [179], dendritic cells [180], and T and B cells [175]. TGF-β is synthesized as a precursor molecule with a signal peptide, a latency-associated peptide (LAP), and the mature peptide of TGFβ. With the removal of the signal peptide, the molecule forms dimeros, which bind to the cell surface of Tregs [181]. TGFβ is activated by the cleavage of LAP by integrins, proteases, and extracellular matrix metalloproteinases [181]. TGFβ plays an important role in the development, function, and maintenance of Tregs. It regulates the genes that maintain the expression of FOXP3 in Tregs, thus maintaining its immunosuppressive function [182]. Owing to crosstalk between TGFβ and other signaling pathways, transcription factors and epigenetic regulators demonstrate the need to understand how these mechanisms function based on multi-omnics and single-cell analysis are required. Wang et al. [175] extensively reviewed the interactions of TGFβ and the TME and signaling-pathway interactions [183].

## 15. Minimal Residual Disease, Latency, and the Immune Reaction

**Figure 5:**
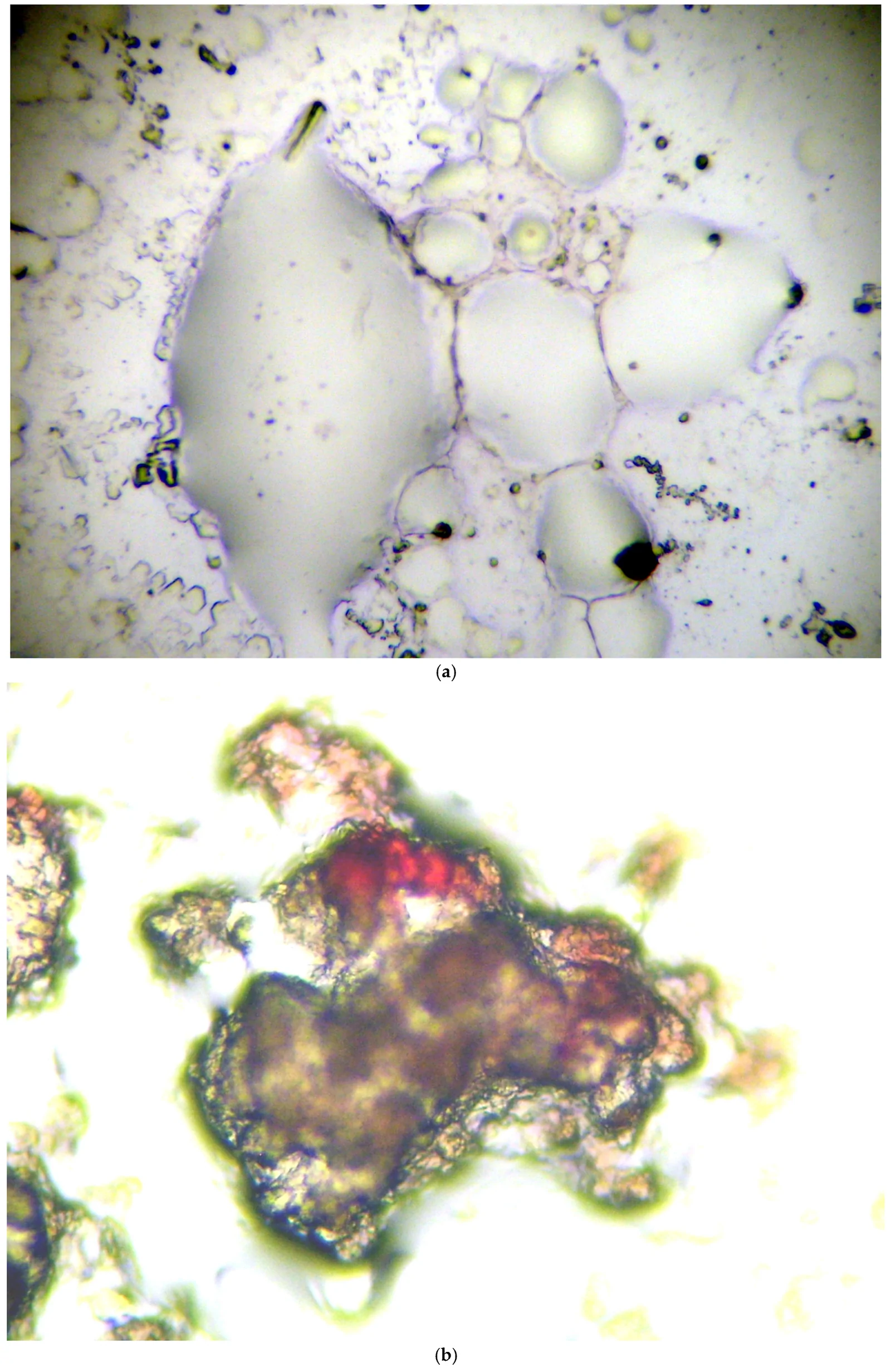
Bone marrow samples, negative for prostate cells and positive for prostate cells, using anti-PSA Immunocytochemistry. (**a**) Bone marrow sample negative for PSA-staining cells. (**b**) Bone Marrow positive for PSA staining cells (red) [114].

Minimal residual disease (MRD) has been defined as small foci of cancer cells located in distant tissues after curative therapy. In prostate cancer, they can be found in some patients after curative radical prostatectomy, implying their dissemination was prior to treatment. Prostate cancer is unique in that it is the only solid tumour whereby treatment failure is defined using a serum biomarker, the PSA, rather than imaging study. Briefly, the initial studies used bone aspirates to detect bone marrow micro-metastasis after an enrichment process using differential gel centrifugation. The collected monocular cells were analyzed using immunocytochemistry. Different monoclonal antibodies were used to identify prostate cells that are not expressed on normal bone marrow cells. These included anti-cytokeratins, EpCAM (epithelial cell anchoring molecules), or anti-PSA. An alternate was the use of PSA or cytokeratin-19 RT-PCR-based detection methods to determine the absence or presence of bone marrow micrometastasis. Although many studies reported an association between the presence of these cells with the clinico-pathological characteristics of the tumour and prognosis, not all studies concluded the same. Variability in detection methods, sample size, the reliability of the antibodies used, pre-analytic, analytic, and post-analytic variables all may affect the conclusions [184,185,186,187,188,189,190]. Effects to standardize the methodology were proposed by the European ISHAGE Working Group in 1999 [191], and later the International Council for Standardization in Hematology (ICSH) in 2015 (Figure 5) [192]. However, the routine use of bone marrow micrometastasis has not been incorporated into prostate cancer treatment guidelines.

With new technologies, automated or semi-automated methods of detection of mcircometasis using blood samples, which is less invasive, and minimal residual disease can be detected. The detection of micrometastic disease may influence tumour staging, cancer cell phenotypes, epigenetic modifications, transcriptional changes, and biomarkers that may indicate the best treatment options [193]. These include the detection of circulating tumour cells (CTCs), circulating tumour DNA, and exosomes, as well as the use of artificial intelligence combined with Big Data bases to identify and monitor changes in minimal residual disease [185]. Although positive samples have a prognostic value, the same problems of method variability, standardization, the need to differentiate between false positives and negatives, and thus comparative analysis of blood samples are not available [193]. A relevant issue is the high cost of these methods, the need of a personal highly trained team in the use of these methods, as well as a multi-disciplined team, and the specific analyzers. A combination of methods may improve the diagnostic and prognostic parameters of patients with prostate cancer without evidence of treatment failure. Tissue biopsy remains the gold standard, but without imaging studies, the important question is where to carry out the biopsy. Each of the aforementioned methods has its drawbacks. Artificial intelligence lacks multicentre clinical validation. CTCs, although they reflect tumour heterogeneity, are difficult to isolate, enrich, and identify. ctDNA, although it possesses a high specificity and sensitivity, lacks large-scale studies and standardized analytical methods due to the instability of the indicators, while exosomes are easy to isolate and enrich, but functional studies are lacking, and there is no standardization of their detection method [193].

## 16. Treatment of Prostate Cancer After Biochemical Failure; The Possible Role of Altering the Immunosuppressive TME

### 16.1. Tumour Microenvironment

After biochemical failure, standard therapies are androgen blockage, first and second line, followed by taxane-based chemotherapy and, finally, immunotherapy [194]. All these treatments are directed at the prostate cancer cells in an asymptomatic patient. Although not directly aimed at the immunosuppressive TME, they do indirectly have effects on the TME.

#### 16.1.1. Androgen Blockage

Briefly, androgen receptor inhibition as monotherapy to treat prostate cancer patients with biochemical failure is a double-edged knife with regards to the immune environment of the TME. There are conflicting reports about its effectiveness in changing the immune response, while for several years, it is able to eliminate the non-resistant cancer cells; it fails as resistant androgen-independent cells survive. There is evidence from studies using primary cancer samples that the androgen-receptor blockade transforms the TME into an inflamed microenvironment within days. Comparing surgical specimens obtained following radical prostatectomy, in patients without prior androgen blockade to those who received three doses of degarelix prior to surgery, there were notable changes in the immune composition of the TME. Activated cytotoxic CD8^+^ T cells and M1-like CAMs were increased. However, this was associated with an increase in Tregs. There was also an upregulation of MHC I and II antigen presentations, as well as a decrease in the levels of CD47 whose expression is associated with immune evasion. Although the results need to be validated, the results implied that the timing and clinical context need to be explored to optimize treatment options [195]. Androgen blockade also decreases the antitumour neutrophil effect against cancer cells found in skeletal metastasis. With the progression of the cancer, there are changes in the neutrophil surface markers and genetic expression, but not the cytotoxicity from an analysis of peripheral blood neutrophils. With use of second-generation androgen blockades, these changes were due to an increased expression of transforming growth-factor beta receptor I. The authors also reported that the use of high-dose testosterone and an inhibitor of the transforming factor resulted in a normalization of the neutrophil antitumour function [196]. The use of low-dose cyclophosphamide, an immunomodulator in itself, plus degarelix versus degarelix without pre-radical prostatectomy in high-risk patients, created a complex response in the immune status of the TME. Both CD8^+^ cytotoxic T cells and Tregs were increased. This implied that adaptive Treg responses could decrease the antitumour immune response [197]. Interestingly, orchiectomy was synergistic with immunotherapy, whereas medical androgen blockade suppressed the effects of immunotherapy. Thus, once again, the timing of the treatment, as well as the dosage of androgen blockage, need to be determined. The inhibition of the CD8^+^ T cell response was caused by the initial priming stage of their differentiation, and not in the phases of reactivation or expansion of T cell phases [198]. In advanced prostate cancer, treatments using immunotherapy have largely showed little activity. However, an androgen-receptor blockade increases the effectiveness of immune checkpoint blockade by increasing CD8^+^ function and decreasing the exhaustion of these T cells. As a result, there was an improved outcome when using PD-1-targeted therapy due to increased IFNγ expression, leading to increased production of cytokines by T-cells [199]. Similarly, Hawley et al. [197], using single-cell RNA sequencing in a Phase II trial comparing chemotherapy versus chemotherapy plus anti-PD-1 immunotherapy, a longitudinal analysis showed dynamic changes in the TME Immune subpopulations changed as a result of treatment and was associated with the clinical outcome. The study reported that a transcriptionally distinct subpopulation of resistant cancer cells increased in treatment-resistant cancer cells. It has also been reported that the use of androgen blockade, in combination with check-point inhibitors, improves immune surveillance and antitumour immune response in these patients [200,201].

#### 16.1.2. Taxanes

Taxanes are standard chemotherapy after failure of androgen blockade. Taxanes not only have a cytotoxic effect on tumour cells via microtubule stabilization via the β-subunit of tubulin, which has a stabilizing function and, as a result, prevents their normal disassembly and chromosome movement during the metaphase and anaphase of mitosis.

As with androgen blockades, taxanes are a double-edged sword. In breast cancer patients, they cause stromal injury, which leads to the end of the latent or dormant period. By injuring stromal cells, protumour cytokines IL-6 and G-CSF produced both in in vitro and in vivo studies regarding dormant cell proliferation. Using single-cell transcriptomes, it was reported that cancer cell stemness and chemoresistance, using stromal organoid models in the analysis of the TME, increased the protumour signaling using a syngeneic mouse model. The role of IL-6 causes cancer cell proliferation in contrast to G-CSF, which causes TME immunosuppression. The use of selumetinib prior to docetaxel therapy, which inhibits the enzymes MEK 1 and 2 and thus the MEK/ERK-signalling pathway, results in the inhibition of proliferation of cancer cells [202]. Taxanes are able to modulate the TME via the immune system, angiogenesis, and metastatic spread [203]. Using in vivo mouse models, it has been reported that taxanes are able to trigger CD8^+^ T cells to release exosomes, which are cytotoxic-causing apoptosis in the cancer cells while not affecting normal cells. Pre-treating with T cell transfer in in vivo models prior to taxane therapy increased the anti-cancer effect of taxanes [204]. Furthermore, taxanes caused pryoptosis, a lytic-programmed cell death in cancer cells via the GSDME-signalling pathway. Single-cell sequencing revealed that GSDME degradation was inhibited by taxanes, thus permitting the recruitment of CD8^+^ cytotoxic lymphocytes and NK cells in prostate cancer [205]. Using a xenograft mouse model, taxanes caused infiltration of the TME of T cells and increased the expression of PD-1 and PD-L1, and this could sensitize mouse cells to anti-PD-1 blockade. Using samples from prostate cancer patients, the combination of a taxane with an anti-PD-1 inhibitor prolonged the PSA progression-free survival [206]. Using tumour-derived exosomes, nanoplatforms or nanofibrous mats loaded with docetaxol, systemic side-effects were minimized but maintained its anti-tumour activity [207,208,209].

#### 16.1.3. Immunotherapy and the Tumour Microenvironment

Since the FDA approval of Sipleucel-T, an autologous cellular immunotherapy for patients with metastatic castration-resistant prostate cancer improved survival and risk of death, as shown in the IMPACT Phase 3 trial in 2010 [210]. A multicentre Phase II trial of its use pre-radical prostatectomy for localized prostate cancer showed changes in the immune cells in the TME. The number of Tregs, CD4^+^ helper T cells, and CD8^+^ cytotoxic T cells were all increased, but the fraction of Tregs only formed a small percentage of recruited T cells. Furthermore, approximately 50% of the CD3 T-cells expressed PD-1 and a higher expression of Ki-67 as compared to pre-treatment samples. There were no changes in the NK cell TME population [211]. At the time, the question was whether combining Sipleucel-T with the checkpoint inhibitors against PD-1/PD-L1, such as ipilimumab, which targets the CTLA-4 receptor, could improve the clinical response. It is important to recall that the TME is dynamic and changes with time, especially the T cell subpopulations [212]. In metastatic lesions, the TME is immunologically “colder” with lower infiltration rates of lymphocytes and activation of NK cells and T-cells, thus reducing the clinical effect of immunotherapies [213]. Combinations of cancer vaccines, which, as monotherapy, have not proved to be successful, and ongoing trials combining them with immune checkpoint inhibitors or monoclonal antibodies are ongoing. Dostarlimab was approved by the FDA for the treatment of patients with a mismatch repair deficiency in castration-resistant metastatic prostate cancer. However, only 1–3% of prostate cancers are positive for this marker [214]. The use of different immunotherapies to target metastatic prostate cancer cells has recently been reviewed [215].

#### 16.1.4. Therapies Against the Immune Cells Found in the Tumour Microenvironment

As previously described, there is an imbalance between the immunosuppressive and anti-tumour immune systems, which is dynamic and changes with time. Therapies directed against these immune cells, either to decrease the immunosuppressive effect or increase the anti-tumour immune response, would appear to be a logical response for treating prostate cancer patients. The challenge is that while the aim is to change the disequilibrium in the TME, the aim is not to change it in normal tissues. Creating an imbalance between the normal immunosuppressive and effector immune responses may lead to serious side effects, which include the Guillain-Barre syndrome, where out of 31 patients, 10 patients developed respiratory failure, and a total of 6 patents died [216]. Autoimmune hypophysitis, which the authors considered as a serious complication, affected a substantial number of patients and that hormone levels should be actively monitored [217]. More serious complications include myocarditis. Although relatively rare, it was seen in approximately 1% of treated patients [218]. The most serious side effect is that of the cytokine release syndrome. Tay et al. [216] reported that these episodes may be repeated with multiple treatments: Grade 3–5 adverse effects had a higher mortality. Of those patients who were retreated with tocilizumab, 50% had fatal outcomes [219]. To put this in perspective, of those 2672 patients treated with checkpoint inhibitors, only 28 patients developed this syndrome, of whom 2 patients developed Grade 5 disease and one patient died. This is in part due to the loss of Treg homeostasis [220]. Preclinical studies have reported that these immune-related adverse effects caused by immune checkpoint inhibitors are due to the inhibition of Tregs. There is an inverse relation between Treg numbers and immune-related adverse effects. Thus, the understanding of these treatments is vital for the production of an anti-tumour response, while at the same time maintaining the immune equilibrium [221]. The suppression of Tregs in in vitro and in vivo mouse models reported that the use of monoclonal antibodies directed against CD25 and CTLA-4 were able to deplete or at least suppress the function of Tregs and thus open up the development of therapeutic options [222].

CAFs play a pivotal role, not only in the reshaping of the mechanical properties of the TME but also in the secretion of exosomes. The development of targeted therapies is difficult owing to the heterogeneity of CAF subtypes. Using single-cell RNA sequencing, four different subtypes of CAFs have been identified [223]. However, their expressed biomarker patterns overlap with those expressed by normal fibroblasts, therefore making it increasingly difficult to identify CAFs and, thus, their specific abilities to support the tumour niche [224]. They also play an important role in prostate cancer by promoting resistance to androgen blockades, radiotherapy, and chemotherapy. Following these therapies, CAFs are not eliminated but enter a latent state while conserving their ability to promote a pro-tumour TME, and it has been described that they are able to increase it [224,225]. Research is continuing to modulate CAF functions and try to enhance the effects of treatment to end the dormant state or to target non-dormant CAFs [225]. Attempts to design targeted anti-CAF therapy in mouse models have had limited success. More recently, the development of organs on chip models gives an opportunity to study the mechanisms of CAF functions and to evaluate targeted CAF therapies in physiologically relevant humanized environments [224]. The proportion of CAF is increased in metastatic prostate cancer as compared to the primary tumour as determined by single-cell RNA sequencing. Using a subcutaneous implantation of cancer cells into a mouse model, and using gene regulatory network analysis, demonstrated that TGF-β and TME remodelling were increased. Those tumours with a higher number of CAFs also demonstrated a higher number of infiltrating inhibitory cells and upregulated levels of immunosuppressive molecules [226]. It has been reported that the use of anti-TNF-β signaling increased the effectiveness of anti-PD-1 immunotherapy in mouse models by upregulating the anti-tumour immune response [226]. The mitochondrial enzyme monoamine oxidase A (MAOA) is involved in the degradation of monoamine neurotransmitters and dietary amines and could possibly be linked to cancerogenesis, owing to the fact that they were upregulated in TME stromal cells. Using a single-cell sequencing database, Zhoa et al. [125] co-cultured CD8^+^ T cells with stromal cells and in vivo using a C57BL/6J tumour model in mice and dual humanized mice where the cell was injected subcutaneously. They reported that the WNT5A-signaling pathway was activated when MAOA was inhibited. This led to the conversion of CAFs to normal fibroblasts and, as a result, a decrease in the immunosuppressive environment [227]. Furthermore, this effect was enhanced when combined with immune checkpoint inhibition causing CD8^+^ T cell activation, possibly presenting a new treatment option. Another study reported on the inhibition of the transmembrane prolyl protease fibroblast activation protein (FAP), which is a surface protein and is highly expressed in CAFs. Using an anti-FAB antibody-drug (momometyl auristatin) conjugate eliminated FAP-expressing cells in vitro and prolonged survival in vivo in mouse xenograft models. There was an increased expression in cancer cells of proinflammatory genes and an increase in the secretion of cytokines IL6 and IL8 by CAFs, as detected by mRNA analysis protein expression. The authors concluded that the eradication of CAFs that express FAP inhibits CAF-cancer cell crosstalk, promoting a proinflammatory response thereby altering the TME and anti-tumour immune response [228].

In a study reported by Hesterberg et al. [226], they identified that asporin secreted by CAFs increased ErbB signaling, causing increased cancer cell migration. They further reported that asporin directly binds to HER3 to form HER2/HER3 dimers, which caused activation of the PIK3 MAPK and calcium pathways using in vitro models. Asporin was detected in the stroma of metastatic prostate cancer cells, which expressed HER2/HER3. Prostate cancer cell proliferation decreased in both in vitro and in in vivo mouse models. In addition, the tumour size was reduced when treated with trastuzumab-deruxtecan, an anti-HER2 antibody drug conjugated, or with patritumab-deruxtecan in HER3 expressing cells. Thus, it possibly has clinical utility in metastatic prostate cancer [229].

By blocking the CXCR2 axis, M2-CAMs can be converted into M1-polarized CAMs; in M2-CAMs, CD36 is highly expressed in neuroendocrine prostate cancer, along with a high expression of CD47 in the cancer cells. By using in vitro prostate cancer cell models and in vivo animal models, it was found that the IL8/CXCR2 pathway via the reprogramming of the Krebs cycle increased acetyl-CoA levels, leading to increased CD47 expression. As a result, cancer cells secreted palmitic acid, inhibiting phagocytosis of normal macrophages. The same pathway was able to recruit CD36^+^ M2-CAMs into the TME. Preclinical studies in animal models showed that by inhibiting the CXCR2 pathway, CAM phagocytic activity was increased and tumour proliferation was reduced, suggesting that CRCX2-targeted therapy may have clinical utility [230,231].

Targeting the CXCR2 axis also reduces MDSCs in the TME and pre-metastatic niche. By comparing the growth and metastasis of breast and melanoma cancer cells in mice who had or did not have deletion of CXCR2 in their myeloid cells, peripheral blood leukocytes and in tumour cells revealed that those mice with deleted CXCR2 resulted in a decreased number of MDSCs and increased levels of CXCL11, which in part was secreted by B1b cells, which were found to be increased in number. B1b cells are able to activate CD8^+^ cytotoxic T cells in the TME, thereby inhibiting cancer proliferation. The same authors reported that treatment using the CXCL2 antagonist SX-682 in mouse models MDSCs was reduced, and B1b cells were increased, as was the activation and recruitment into the TME of CD8^+^ T cells [232]. Various studies using different inhibitors to reduce the number and activity of MDSCs resulted in an increased anti-tumour immune response by CD8^+^ T-cells. This includes the use of monoclonal antibodies to cause the inhibition of CCCL2-CCR2 axis using anti-CCCL2 antibodies or antagonists, which showed promising results in preclinical studies [233,234]. Disappointingly, in a phase II trail, the use of monoclonal antibody carlumab, an anti-CCL2 antibody, in patients with metastatic castration-resistant prostate cancer revealed a rapid increase in circulating free CCL2 [235]. An important role in cancer progression is the CCR5-CCL5-signaling axis: activation of this pathway induces proliferation and migration of cancer cells [236]. Inhibition of the colony-stimulating factor 1 receptor, which is only expressed in monocytes and macrophages, is currently being investigated using emactuzumab in combination with chemotherapy or immunotherapy in patients with cancer [237]. Its use in combination with atezolizumab produced an overall response rate of between 5.6% and 12.5%, depending on pre-treatment factors [238]. Various in vitro studies have identified molecules that inhibit the immunosuppressive TME and, as a result, improve the anti-tumour immune response. Galiellalactone inhibits STAT3 and thus the production of MDSCs by prostate cancer cells, which decreases the immune-suppressive immune system [239]. Targeting EP4, as determined by single-cell analysis, acts as a target for T cell infiltration, and it is inhibited by YY001 both in in vitro and in vivo models by changing the profile of chemokines. When combined with anti-PD-1 checkpoint inhibition, it increased CD8^+^ cytotoxic T cell function and their accumulation in the TME, converting the “cold” TME into a “hot” TME, which in in vitro animal models resulted in an increased reduction in tumour size and overall survival [240]. Finally, an inhibitor of histone deacetylase, CN133, reduced MDSC infiltration and was used in combination with anti-PD-1 checkpoint inhibition to increase the anti-tumour response in models using allograft subcutaneous tumour models. However, this was only seen in soft tissue and not in bone metastasis [241].

On the other side of the coin, therapies to increase the anti-tumour immune system have been investigated. The humanized anti-NKG2A antibody, monalizumab, increased the anti-tumour immune response by increasing the activity of both NK cells and CD8^+^ cytotoxic T cells when used in combination with anti-PD-1 inhibition. A phase II trial of monalizumab, combined with cetuximab in patients with previously treated squamous head and neck cancer, achieved an overall response rate of 31% [242]. The monoclonal antibody ADWA-11 blocks the role of αvβ8 integrin, which inhibits anti-tumour immunity, especially when used in combination with immunomodulators as seen in in vitro models [243]. The use of the anti-CD38 monoclonal antibody daratumumab or the CSF-1R inhibitor edicotinib before radical prostatectomy was investigated. However, there were no changes in the serum level of PSA or the achievement of a complete pathological response, with neither of the two treatments inducing infiltration of T cells into the TME [244]. Galectin-1 levels have an association with advanced stages of prostate cancer and have the potential to induce apoptosis of T cells. The use of LLS30 in vitro inhibited the effect of galectin by binding to CD45, which was expressed by prostate cancer cells and thus the inhibition of T cell apoptosis. Using RNA sequence analysis, it was shown that LLS30 enhanced the anti-tumour immunity via cytotoxic CD8^+^ T cells [245]. The new method of irreversible electroporation of prostate cancer cells is associated with a decreased immunosuppressive TME. Electroporation involves using needle-like electrodes to deliver short, high-voltage electrical pulses around the prostate gland using imaging guidance. The strong electrical field disrupts the cells’ lipid membrane, causing irreversible membrane damage and cell death via apoptosis or necrosis. Damage to the surrounding tissues is reported as minimal, and it also stimulates the anti-tumour immune response. In a small study of 30 patients, irreversible electroporation was tested against robot-assisted radical prostatectomy using the IFγ enzyme-linked enzyme assay. The immune responses in both groups were analyzed over time. Electroporation decreased the number of Tregs while increasing the number of activated cytotoxic T cells, which reached its maximum peak 14 days after treatment. The authors suggested that these results could be improved by using a combination with CTLA-4 checkpoint inhibitors [246].

## 17. Discussion

The development of multi-omnics has given the opportunity to study in-depth changes in the cancer cells, immune function, proteins, and lipids as compared to normal cells and enables the detection of changes that occur over time or after treatment. This presents the same challenges as with the detection of MRD. In the case of MRD, the use of bone marrow samples, CTCs, ctDNA, or, more recently, exosomes have been shown to be associated with a worse prognosis before and after treatment. However, the main failure was the lack of standard in the methods used, in the pre-analytical, analytical, and post-analytical phases, although guidelines were presented. In the case of ctDNA, the process is automatized, and in gene mutations such as KRAF, the RAS gene family can be identified and thus help in the clinical decision of the best treatment option. Its use can be found in the management of colon cancer, lung cancer, and melanoma to select the best treatment options. In the case of prostate cancer, where failure is defined by a serum biomarker, the PSA in a non-symptomatic patient treatment. Choices should have minimal toxicity to maintain the quality of life of the patient. This raises the question of “should MRD be treated and with which therapy?” Early studies using an androgen blockade was shown to reduce the micro-metastatic burden, but not all cells were eliminated, leaving the resistant cells to possibly proliferate and later produce biochemical failure [247,248,249]. Newer investigation using lymph nodes resected at the time of radical prostatectomy revealed that PSA mRNA levels correlated with routine immunocytochemistry. However, using qRT-PCR showed that PCA3 expression could discriminate the presence of lymph node micrometastasis and could be an improvement with respect to PSA gene activity [250]. Using the DU145v knock-out cell line in mouse models, it was reported that the knock-out of 15 selected genes caused an increased potential for the proliferation and metastasis of DU145 cells [248]. The use of artificial intelligence has been increasingly used in prostate cancer studies and improved the detection of lymph node micro-metastasis as compared with standard pathological evaluation of specimens [249]. Finally, although the use of qPCR of PSA-mRNA outperformed standard histology in the detection of lymph node micro-metastasis independent of biochemical failure during a 5.4-year period, it was not better than standard histology in predicting biochemical failure [250].

With the development of multi-omnics, the challenges are much greater, from genomics analysing the gene sequence, to epigenomics analysing modifications to the DNA in terms of the states of methylation and hydroxylation, transcriptomics, proteinomics, and metabolomics. These methods investigate the tumour as an ecosystem, such as the interaction of all the components under study; for example, proteins. With advent of new technologies, such as next-generation sequencing, microassays, whole genome sequencing, whole exosome sequencing, PCR in real time or digital, mass spectrometry, microRNAs, and promotors of hypermethylation, this generates an enormous amount of data. Not every method is a “one fits all” solution, but each omnic subtype requires a different methodology. The enormous amount of data has to be stored, and selective data are used in each analysis depending on the question to be asked. The use of artificial intelligence and machine learning has facilitated this task. However, the whole database needs a computation system with a capacity to store and process this information and should be linked to the different centers of investigation. Each method may require different types of samples, fresh, frozen, or paraffin-embedded tissues. There needs to be a multidiscipline team of well-trained professionals to analyse the data, but at the end, the final decision is clinical.

For example, analysing epigenetic changes can give important information in a clinical setting. Methods that are used to detect the hypo or hypermethylation of DNA, microRNAs, long non-coding RNAs, and circular RNA include microarrays.

Other more widely used methods are quantitative PCR to analyze microRNAs, or to identify genes with methylation changes using methyl-specific high-resolution milking. MethylLight is a method based on quantitative PCR using specific probes for regions methylated or not methylated. Epigenetic markers can be analyzed using liquid biopsies, fresh or frozen tissues, or using paraffin-embedded tissue samples with high sensitivity and specificity. There are commercial kits to analyze these epigenetic changes for patients with colorectal and breast cancer, which provide relevant clinical information. In colorectal cancer, using RT-qPCR on paraffin-embedded tissue samples, the epigenetic marker miR-31-3p can be identified. A methylation change is associated with the benefit of using anti-EGFR versus anti-VEGR therapy. Meanwhile, in breast cancer using paraffin-embedded tissue samples or ctDNA and the MethylLight method to identify DNA methylation of PITX2, a change in the methylation status is associated with the response of the cancer to anthracycline based chemotherapy.

## 18. Conclusions

When prostate cancer develops, it forms a small part of the normal stromal microenvironment. By the aforementioned mechanisms, it is able to transform this microenvironment into an immunosuppressive tumour microenvironement, converting normal stromal cells into cancer-associated cells. It also is able to inhibit the anti-tumour immune response, which occurs early in the disease even before “curative” therapy, which explains the presence of minimal residual disease. By a better understanding of immunosuppressive mechanisms, the interactions between the different players in the tumour ecosystem, as well as multi-omnic changes using single-cell analysis, it may be possible to design new therapies to reverse the immunosuppressive process and stimulate the anti-tumour immune response. This potentially may improve the prognosis of the patient, and if validated and used in the context of a validated MRD analysis, may have a higher potential to eliminate or at least maintain remaining cancer cells in a dormant state as the total tumour load is at a minimum.

## Data Availability

No new data were created or analyzed in this study.
